# Molecular solubilization of fullerene C_60_ in water by γ-cyclodextrin thioethers

**DOI:** 10.3762/bjoc.8.188

**Published:** 2012-09-28

**Authors:** Hai Ming Wang, Gerhard Wenz

**Affiliations:** 1 Organische Makromolekulare Chemie, Saarland University, Campus Geb. C4.2, D-66123, Saarbrücken, Germany

**Keywords:** C_60_, cyclodextrins, dynamic light scattering, particle size, solubilization, UV spectrum

## Abstract

Various hydrophilic γ-cyclodextrin (CD) thioethers, containing neutral or ionic side arms were found to form molecular disperse solutions of C_60_ in water reaching concentrations of 15 mg/L. Equilibrium state was approached after seven days without the use of organic cosolvents. The 1:2 stoichiometry of the C_60_/γ-CD thioether complexes was demonstrated by a parabolic phase-solubility diagram. In contrast, native γ-CD forms nanoparticles with C_60_. Particle sizes of C_60_ were determined by dynamic light scattering.

## Introduction

Since the first spectroscopic discovery of buckminsterfullerene, C_60_, by Kroto, Heath, Curl and Smalley in 1985 [1], and its first macroscopic synthesis by Krätschmer et al. in 1990 [2], this third allotropic modification of carbon has been the subject of more than 10,000 publications giving rise to many interesting potential applications both in the biomedical field [3–5] and materials science [6–9].

A good solubility of C_60_ in water is especially required for biological applications; however, this is not the case at all. Solubility of C_60_ was estimated to be as low as 10^−8^ ng/L, equivalent to 10 C_60_ molecules per millilitre of water [10–11]. Therefore, hydrophilic derivatives of C_60_ have been synthesized and utilized for the inhibition of therapeutically important enzymes, such as HIV-1 protease [12], for the prevention of bacterial growth [13–14], or for photodynamic therapy of cancer by scission of DNA [3]. Despite these successes, there are still several issues relating to the chemical modification of C_60_. The regioselectivity of derivatization is difficult to control [15], and derivatization reduces aromaticity, which leads to a change of the distinct electronic and photonic properties of C_60_.

Dispersions of C_60_ nanoparticles in water have been discussed as alternatives for molecular solutions. Such C_60_ dispersions are generally obtained by the so-called solvent-exchange method [16–17], where C_60_ is dissolved primarily in an organic solvent, such as benzene, THF, or acetone, and afterwards diluted with water. After evaporation of the organic solvent, clusters of C_60_ in water remain, which are temporarily stable. The biological activities of those dispersions strongly increase with decreasing size of the C_60_ nanoparticles [13]. Also micellar dispersions of C_60_ in water stabilized by detergents such as Tween, Triton, or SDS are known [18]. Applications of these C_60_ dispersions are hampered by the toxicities of the employed organic solvents or surfactants.

**Figure 1:**
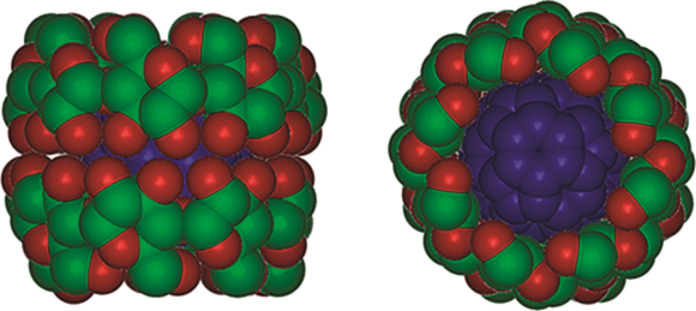
Space-filling model of the most stable complex between γ-CD and C_60_ with 1:2 stoichiometry, calculated in vacuo. Reprinted with permission from reference [29]. Copyright (2010) American Chemical Society.

The most successful strategy to carry the extremely hydrophobic C_60_ molecule into water is the use of appropriate water-soluble carriers that can form host–guest complexes, such as calixarenes [19–20] and cyclodextrins (CDs) [21–22]. CDs are cyclic oligosaccharides consisting of six, seven, eight or more glucose subunits connected through α-1,4 glycosidic linkages, called α-, β-, and γ-CD, respectively. CD molecules resemble truncated cones comprising a hydrophilic outer surface and a relatively hydrophobic cavity [23–25]. CDs form water-soluble inclusion complexes with many hydrophobic or amphiphilic guest molecules [26], mainly driven by hydrophobic interactions [27]. Among the commercially available CDs, γ-CD with a clear width of *d* = 0.74 nm [28] is only large enough to partially accommodate C_60_, which has a still greater van der Waals diameter of 1.0 nm [2]. Molecular dynamics studies strongly favour a sandwich-like structure of the complex, in which two γ-CD molecules tightly interact through hydrogen bonds between their secondary rims and in which C_60_ is situated in the middle at the widest sites of both CDs (see Figure 1, [29]).

Andersson et al. reported that C_60_ formed a water-soluble 1:2 inclusion complex with γ-CD after heating under reflux in water [21]. Mixing of an aqueous solution of γ-CD with a methanolic solution of C_60_ led to a C_60_ dispersion with a concentration of ca. 70 mg/L [30]. Even higher concentrations of up to 1 g/L were reached by high-speed vibration milling of C_60_ in aqueous solutions of γ-CD [31]. The main drawbacks of these aqueous systems are still (a) their lack of stability, leading to crystallization of the C_60_ complex after some days [31]; and (b) their pronounced tendency to form nanoparticular aggregates [21,32], both of which limit their practical application.

**Scheme 1:**
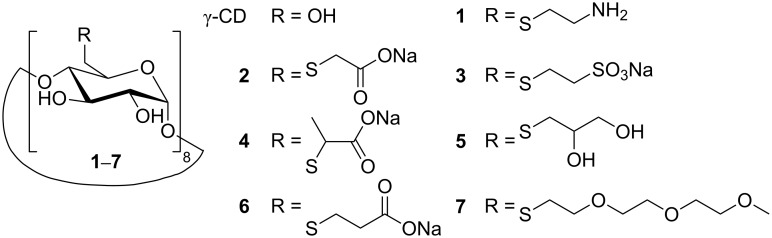
Chemical structures of γ-CD and γ-CD thioether **1**–**7** used to solubilize C_60_ in water.

Recently, we developed a new class of highly water-soluble per-6-deoxy-thioethers of β- and γ-CD (>20% w/w), which showed exceptionally high solubilization abilities for several aromatic molecules, such as anthracene and acenaphthylene, in water [33–35]. In this work, we investigated solubilization of C_60_ by these γ-CD thioethers (compounds **1**–**7** in Scheme 1) with the hope of achieving high concentrations of solubilized C_60_. The hydrophilic substituents at the primary face of γ-CD should increase solubility and avoid aggregation, because they cannot form intermolecular hydrogen bonds like the primary hydroxyls of native γ-CD.

## Results and Discussion

### UV–vis spectra of C_60_ solutions

**Figure 2:**
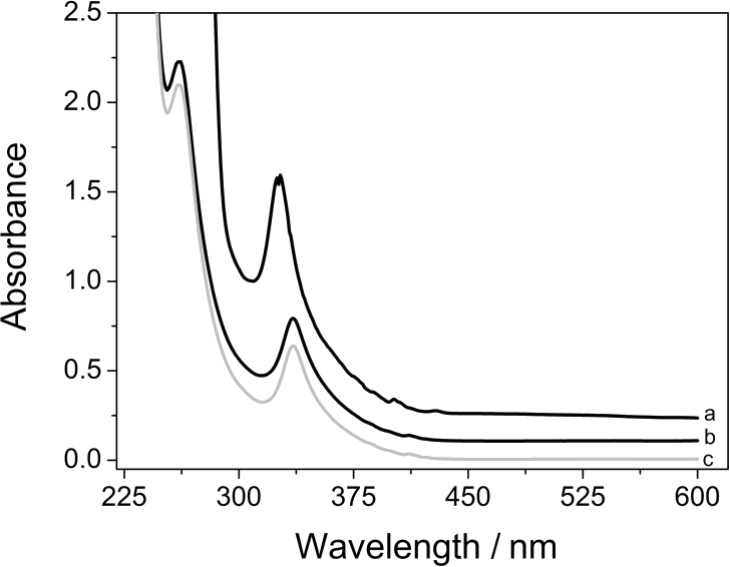
UV–vis spectra of (a) C_60_ solution in THF, (b) aqueous solutions of C_60_ with 6 mM γ-CD thioether **5** before centrifugation, and (c) after centrifugation (13,000 rpm) for 60 min. The absorbance intensities of (a) and (b) are shifted by 0.2 and 0.1 AU, respectively.

Powdered C_60_ was stirred at 25 °C in 6 mM aqueous solutions of γ-CD and γ-CD thioethers **1**–**7** giving rise to clear, dark yellow solutions of C_60_, which show a narrow absorption band at λ_max_ = 335 nm quite similar to that of a solution of C_60_ in THF (λ_max_ = 327 nm), shown in Figure 2. Extensive centrifugation caused only a small reduction of signal intensity (less than 8%, Figure 2), which clearly precludes the existence of aggregates, which would otherwise sediment to the bottom. Therefore the molar concentration of C_60_ could be calculated by using the known molar extinction coefficient of C_60_ molecularly dissolved in *n*-hexane at 328 nm, i.e, ε = 52,000 M^−1^·cm^−1^ as published previously [36].

### Dissolution kinetics

**Figure 3:**
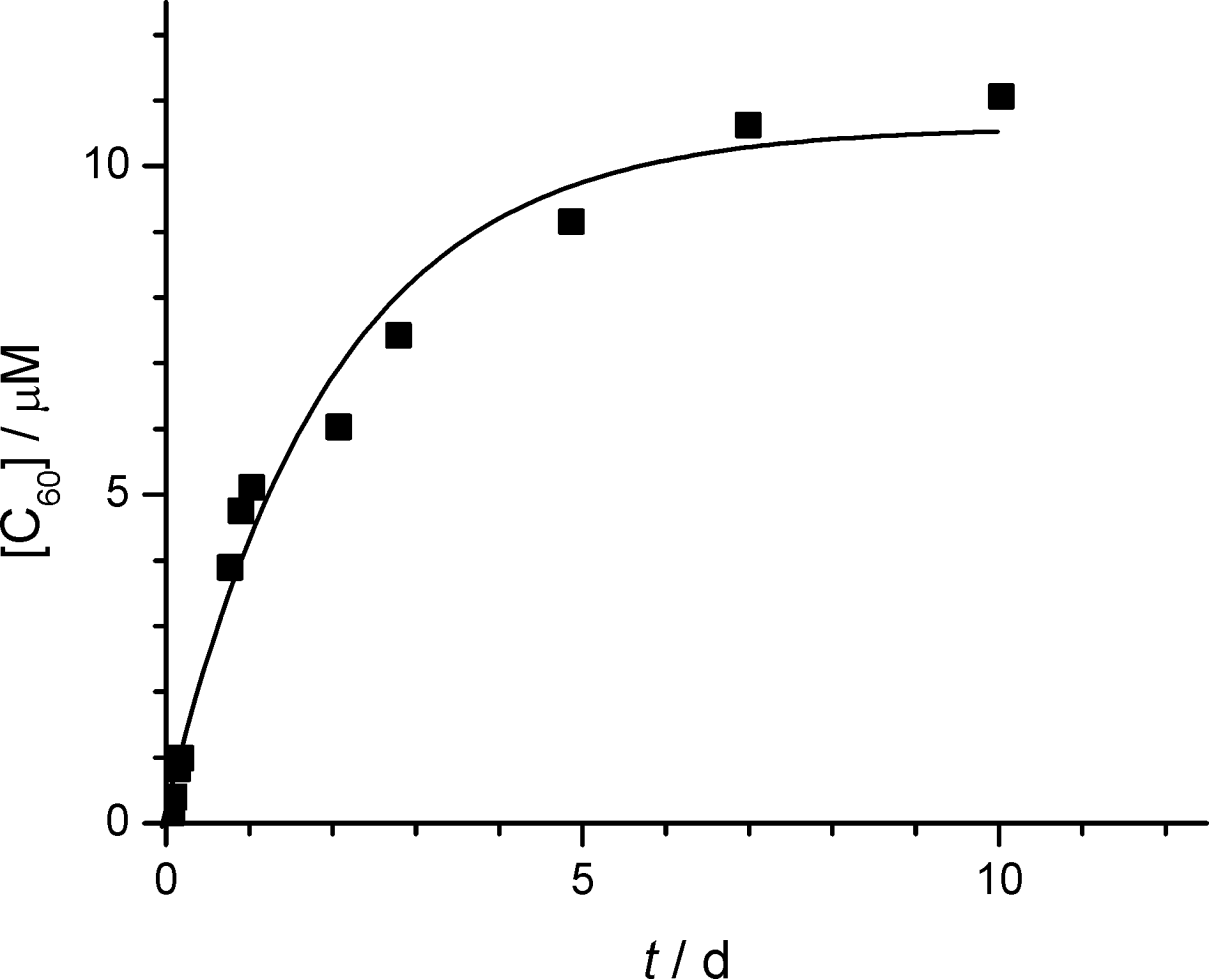
Isothermal kinetics of the dissolution of C_60_ in the presence of 10 mM CD **7** in water. Curve: best fit of first-order kinetics *k* = 0.5 d^−1^ = 0.021 h^−1^.

After the dissolution of C_60_ by γ-CD thioethers had been demonstrated, we were interested in how long it takes to reach equilibrium, within experimental error. Therefore a thinly casted film of C_60_ was incubated with an aqueous solution of γ-CD thioether **7** at 50 °C and stirred according to Kuroda et al. [37]. The slow increase of C_60_ concentration was monitored by the increase in absorption intensity at 335 nm. The observed first-order dissolution kinetics of C_60_ came nearly to an end after 7 d, as shown in Figure 3. The obtained rate constant, *k* = 0.021 h^−1^ was somewhat higher than the one already found for native γ-CD, *k* = 0.011 h^−1^, which also showed first-order dissolution kinetics [37].

The observed simple first-order kinetics was puzzling for us, because the concentration of the CD host does not go down significantly (<<1%) over the course of the dissolution of C_60_. Therefore other reasons for the observed continuous decrease of dissolution rate had to be found.

**Scheme 2:**
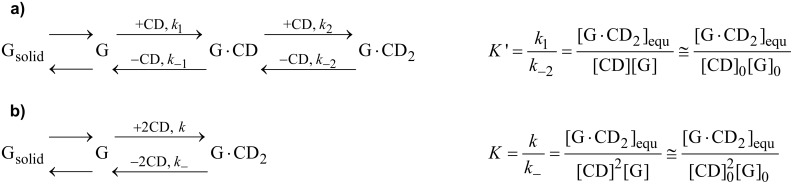
Mechanistic description of the two possible mechanisms for the complexation of C_60_ (G) by CD hosts, in (a) two steps, and (b) one step.

Dissolution of C_60_ can be described by a two-step process, originally proposed by Kuroda et al. [37]. Alternatively, a one-step process, in which a C_60_ molecule is trapped by two CD rings at the same time, appeared reasonable to us. Both models are shown in Scheme 2.

The two-step model (a) employs a slow, rate-determining complexation of the guest C_60_ by the first CD molecule and a fast further complexation by the second. The second step is much faster than the first due to the strong stabilization exerted by multiple hydrogen bonds between both CD rings. For the back process, the first dissociation step should be slow and rate determining, because of the necessary cleavage of these intermolecular hydrogen bonds. These assumptions lead to an apparent equilibrium constant *K*′ in which the CD concentration is only present in first order. The initial rate of complexation is predicted to be proportional to CD concentration, which was already experimentally found for native γ-CD [37].

On the other hand, the one step model (b) leads to the classical binding constant *K*. The initial complexation rate should be proportional to the square of the host concentration, as shown in Supporting Information File 1.

Both models have in common that the formation of the complex follows pseudo-zero-order kinetics whereas dissociation follows first-order kinetics. Consequently, the integrations of the rate equations for both models (described in Supporting Information File 1) lead to the same final kinetics (Equation 1), a simple first-order equation converging to the equilibrium solubility of the guest [C_60_·CD_2_]_equ_ in agreement with the observed experimental data. According to both new models, the obtained rate constants *k*_−2_ and *k*_−_*_,_* respectively, are not due to formation of the complex as proposed previously [37], but due to its dissociation.

[1]
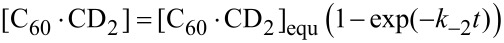


### Dependence of the equilibrium concentration of C_60_ on the host and its concentration

Equilibrium concentrations of C_60_ in aqueous solutions of 6 mM γ-CD and γ-CD thioethers **1**–**7**, determined from the absorptions (λ_max_ = 335 nm) after being stirred for 7 d, are listed in Table 1. C_60_ concentrations obtained with γ-CD thioethers were up 35 times higher than the one obtained with native γ-CD, in accordance with previous results for other hydrophobic guests [38]. The improved solubilization potential of the thioethers was attributed to the higher hydrophobicity of sulfur compared to oxygen. The highest concentration of C_60_ in water, 14.9 μM, was found for CD derivative **5** with attached neutral diol substituents. In general, neutral γ-CD thioethers **1**, **5**, and **7** performed better than the anionic ones **2**, **3**, **4**. Coulomb repulsion between the anionic groups in between the two CD molecules was held responsible for the reduced binding affinity. Astonishingly, the amino derivative **1** also showed a high solubilization potential, which may originate from the addition of the amine to a double bond of C_60_, as was already observed by Geckeler for other amino compounds [39].

**Table 1:** C_60_ concentration in 6.0 mM aqueous solutions of γ-CD and γ-CD thioethers **1**–**7**.

host	[C_60_] (μM)

γ-CD	0.4
**1**	10.3
**2**	2.9
**3**	5.3
**4**	2.1
**5**	14.9
**6**	7.6
**7**	9.3

**Figure 4:**
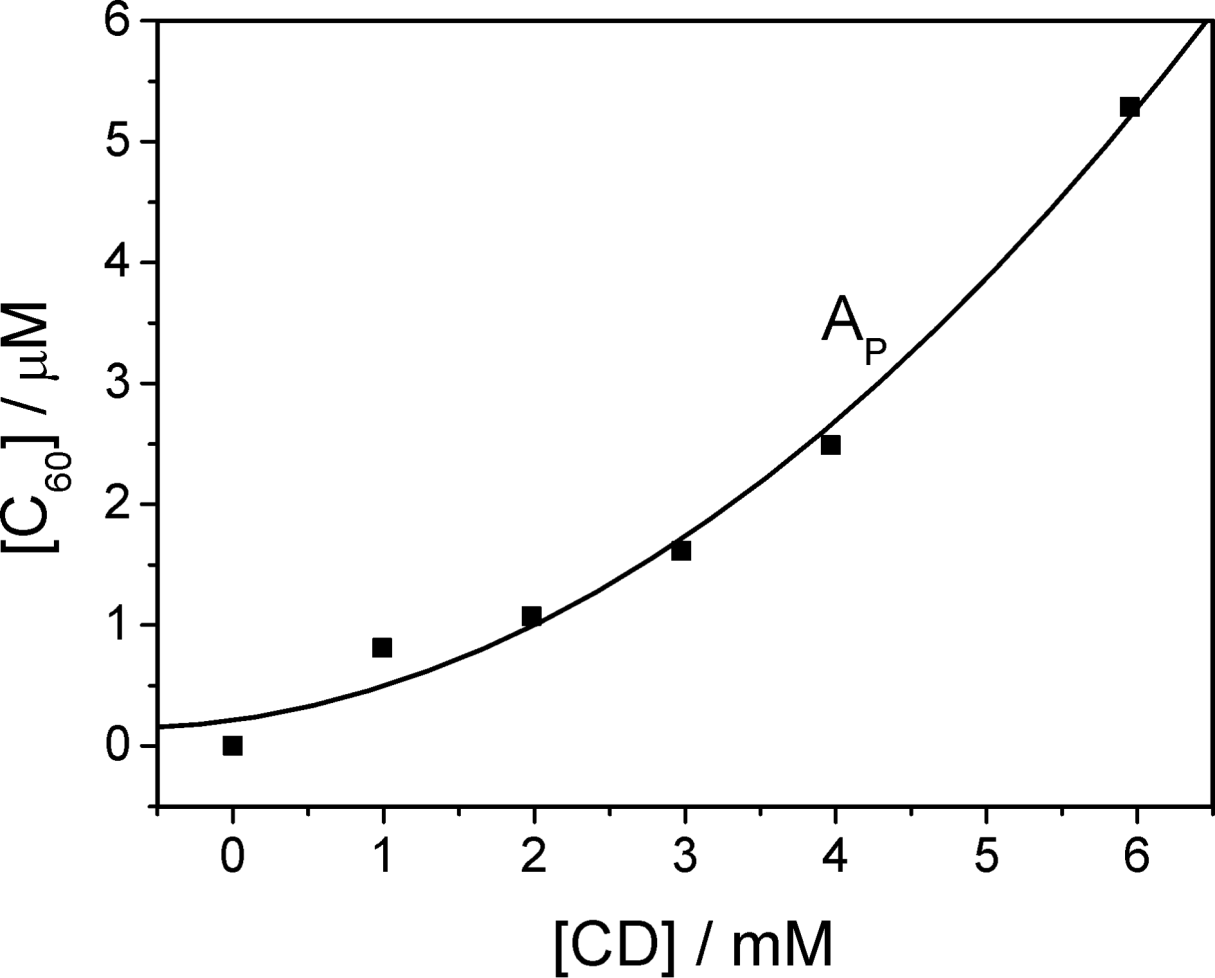
Phase-solubility diagram of C_60_ in aqueous solution in the presence of CD **3**.

The phase-solubility diagram of C_60_ in the presence of γ-CD thioether **3**, according to the method established by Higuchi and Connors [40], was obtained by plotting the concentration of the dissolved C_60_ versus the concentration of the host, as depicted in Figure 4. The observed parabolic A_P_-type phase concentration dependence is typical for the formation of complexes with 1:2 stoichiometry [40–41]. The equation for the best fit [C_60_] = 10^−3^(0.21 + 0.17[CD] + 0.11[CD]^2^) indicates that the two-step process (first order in [CD]) as well as the one-step process (second order in [CD]), discussed above, contribute to the dissolution of C_60_.

### Preparation of C_60_ complexes with the aid of organic solvents

Because equilibration took a long time (7 d) and occupancies of the γ-CD thioethers are still low (<0.3%), organic solvents, such as toluene, DMF or CS_2_ were added to the aqueous solutions of γ-CD and γ-CD thioether **5**, in the hope of accelerating and improving the dissolution of C_60_ as reported by Murphy et al. [42]. The resulting solutions were characterized by UV–vis spectroscopy. The saturation concentrations of C_60_ are listed in Table 2.

**Table 2:** C_60_ concentration in 6.0 mM aqueous solutions of γ-CD and γ-CD thioether **5**.

CD	[C_60_] (μM)			
	procedure **a** water	procedure **b** water/toluene	procedure **c** water/DMF/toluene [42]	procedure **d** water/CS_2_

γ-CD	0.4	7.5	4.9	10.8
**5**	14.9	21.4	6.3	18.6

**Figure 5:**
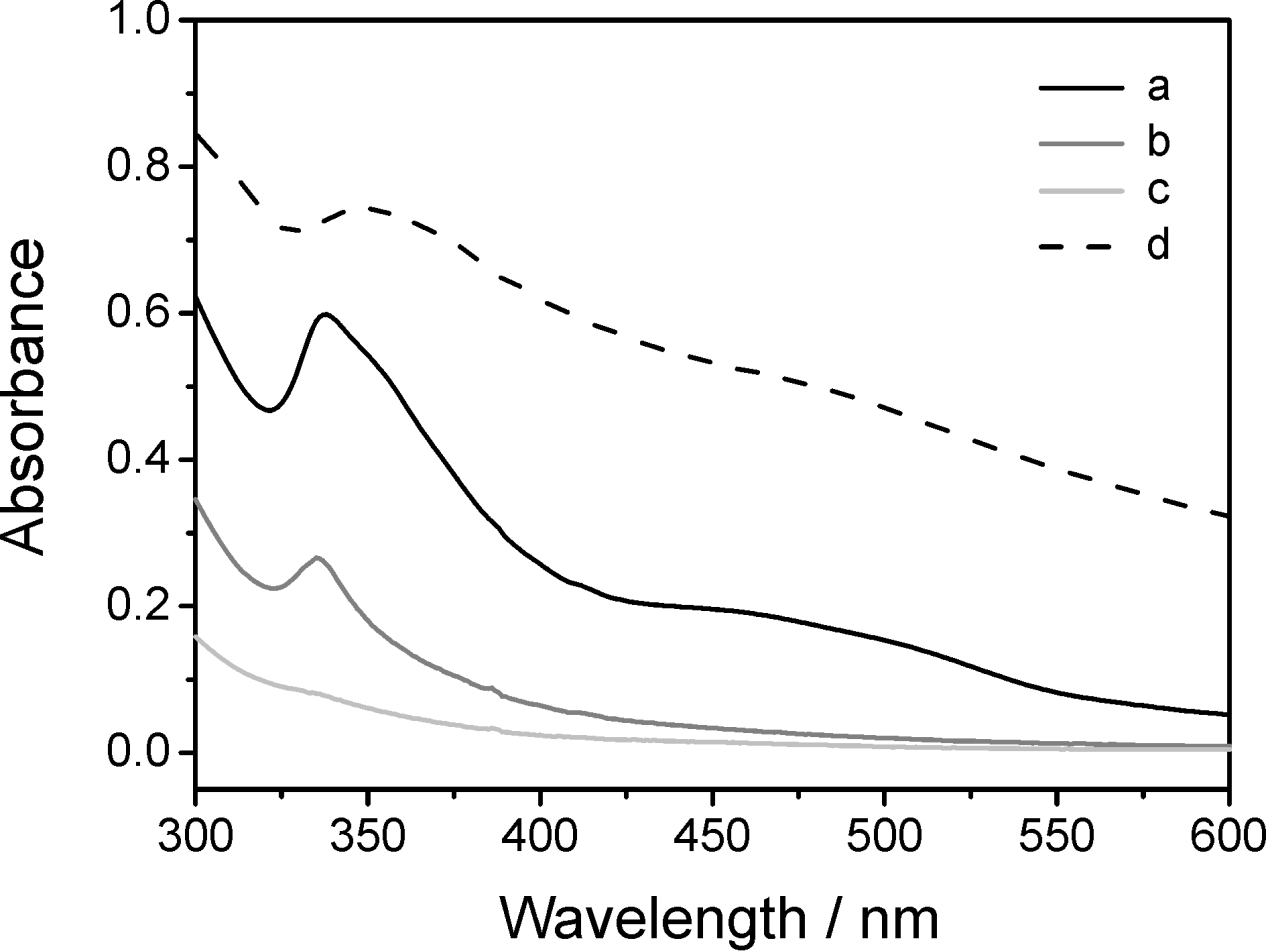
UV–vis spectra of the water solution of C_60_ produced by stirring C_60_ in 6mM γ-CD solution in DMF/toluene (v/v 1:1) at rt for 7 d, followed by dissolution of the resulting complex in water after evaporation of the solvents (procedure **c**): (a) before filtration, (b) after filtration, and (c) after centrifugation; (d) *n*C_60_ made from THF [43] before filtration.

Indeed, for native γ-CD much higher C_60_ concentrations could be achieved in water with the aid of organic solvents. But careful examination of the UV–vis spectra of the C_60_ solutions produced according to procedure **c** [42] before filtration, after filtration, and after centrifugation, as shown in Figure 5, revealed significant differences. The unfiltered solution of the C_60_/γ-CD complex showed an additional absorption maximum 475 nm, which is typical for C_60_ nanoparticles (*n*C_60_) such as those formed by dilution of a solution of C_60_ in THF with water [16,43]. After centrifugation nearly no C_60_ was left. Consequently, apparent improvements of the solubilization obtained with the aid of organic solvents were mostly due to the formation of nanoparticulate dispersions. Since these organic solvents are also hazardous for most cells, applications in biomedicine are prohibited. Therefore, organic solvents were avoided for the dissolution of C_60_ by γ-CD thioethers, because they also did not improve the solubilization process significantly.

### Investigation of aggregation by dynamic light scattering

Since aggregation of CDs and CD inclusion compounds was already found in previous work [44–45], dynamic light scattering (DLS) investigations were performed to check for any aggregation during solubilization of C_60_ by γ-CD thioethers. DLS is a relatively fast method for the determination of the particle size distributions of proteins, polymers, micelles, and nanoparticles [46]. In particular, DLS is able to distinguish between a homogenous molecular solution and a dispersion of aggregates [47–48].

**Figure 6:**
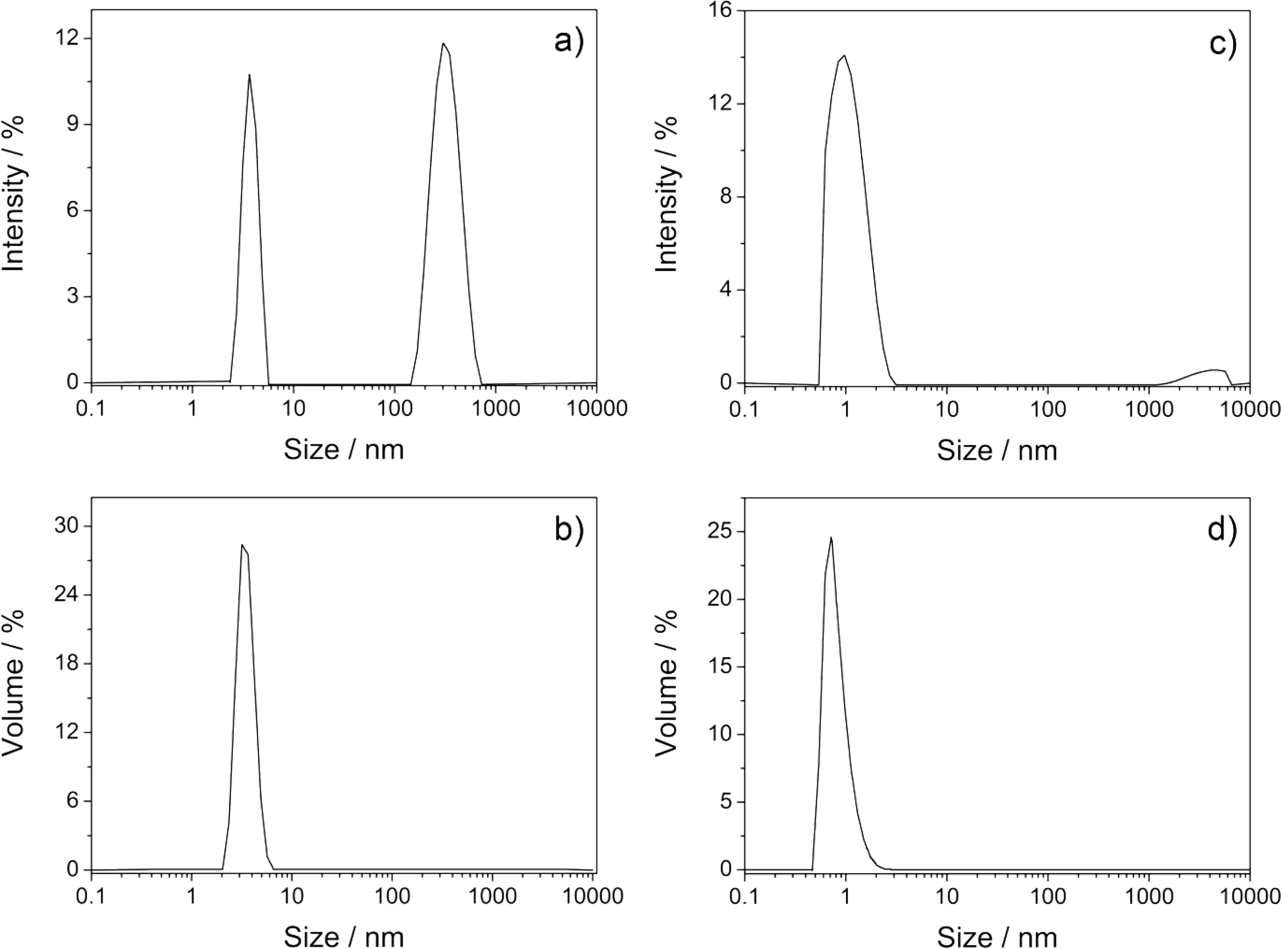
Size distribution of the molecular solution of C_60_ with 6 mM CD **5** in water at 25.0 °C: before centrifugation (a) by intensity, and (b) by volume; and after centrifugation (c) by intensity, and (d) by volume.

The size distribution of the solution of C_60_ in 6 mM γ-CD thioether **5** before centrifugation, shown in Figure 6, comprised two peaks at particle sizes of 3 and 300 nm. The first peak was attributed to the molecular CD/C_60_ complex, the second to aggregates of it. Since the intensity of the scattered light increases with the sixth power of the particle size, the content of aggregates is highly overestimated [45]. For getting the right picture, this intensity profile had to be transformed to the volume distribution profile, by using the Mie theory [49]. In the resulting volume distribution profile (Figure 6b) only one peak remains, which corresponds to the molecular complex. Consequently, this solution mainly consists of molecularly dissolved C_60_. This finding is in accordance with the negligible decrease of C_60_ absorption caused by centrifugation, shown in Figure 2.

**Figure 7:**
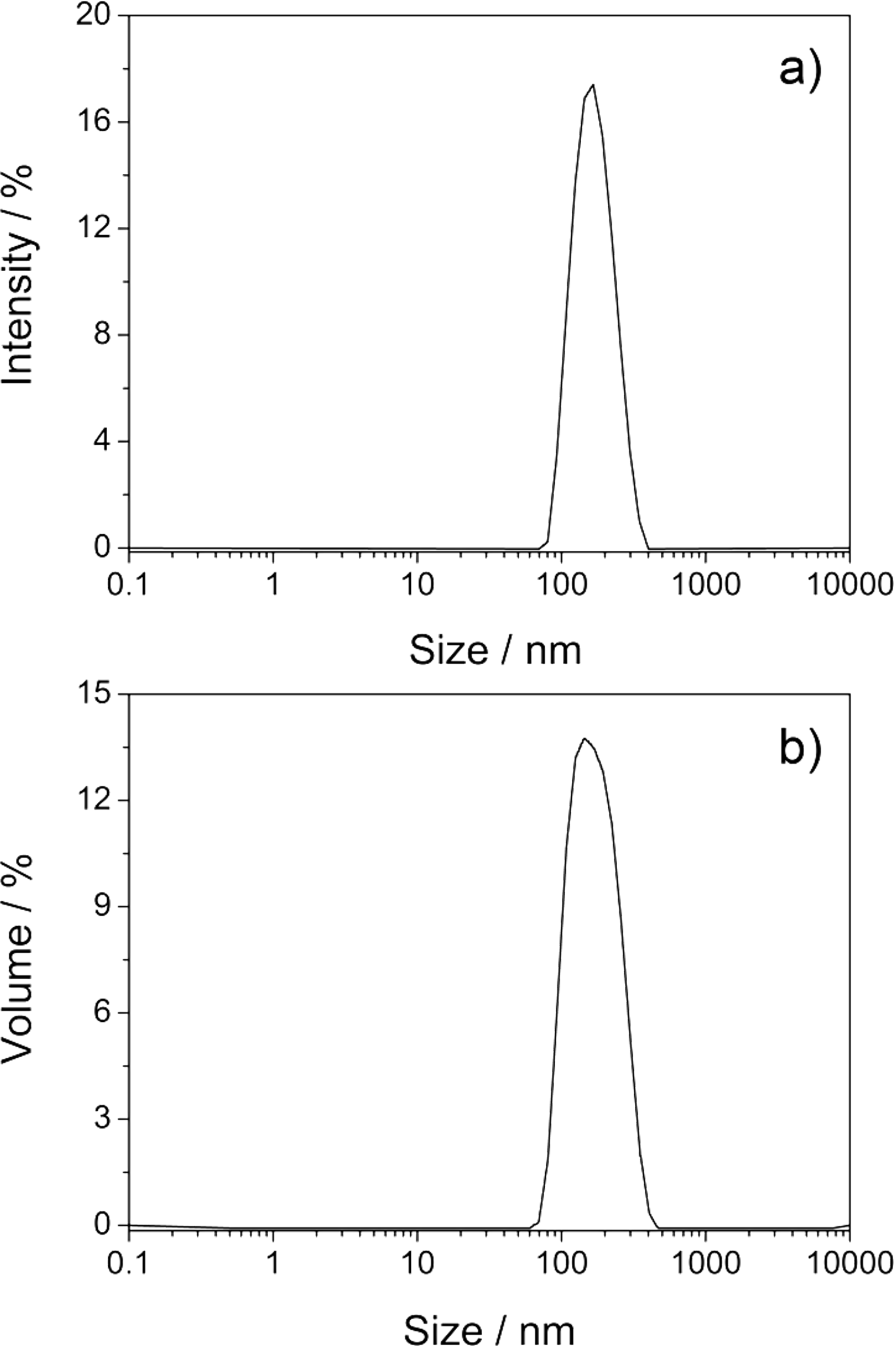
Size distributions of the aqueous C_60_ dispersions after filtration, produced by stirring C_60_ in 6mM γ-CD solution in DMF/toluene (v/v 1:1) according to procedure **c**) [42]: (a) by intensity and (b) by volume.

After centrifugation (13,000 rpm) for 60 min, shown in Figure 6c, only a sharp peak at 1 nm was observed for both the intensity and the volume size distributions, which demonstrates the validity of the DLS measurement. For comparison, the corresponding intensity and volume size distributions of a freshly prepared C_60_ solution in the presence of 6.0 mM native γ-CD according to procedure **c** [42] (Figure 7) only showed one peak at a diameter of 166 nm, very similar to *n*C_60_ prepared from THF. Consequently, the DLS investigations confirmed our previous finding that solubilization of C_60_ in water with the aid of organic solvents results in dispersions of C_60_ nanoparticles.

## Conclusion

Neutral γ-CD thioethers are especially well suited as solubilizers for C_60_. Sandwich-like 1:2 complexes are formed at room temperature without the necessity of adding organic cosolvents. These complexes show a much lower aggregation tendency than the corresponding ones of native γ-CD. Molecular solubilization of fullerene C_60_ in water, reaching concentrations as high as 15 μM, was achieved in the presence of 6.0 mM of a γ-CD thioether. The resulting aqueous molecular solutions of C_60_ free of toxic organic solvents will hopefully find interesting applications in biomedicine, such as in photodynamic therapy or HIV-protease inhibition.

## Experimental

### General

Unless otherwise stated, all chemicals were used as received. Powdered fullerene C_60_ (> 99%) was purchased from Sigma Aldrich. Teflon syringe filters from Roth, Karlsruhe, Germany (0.45 μm) were used to remove insoluble material before UV–vis spectrophotometric analysis. UV–vis spectra of aqueous samples were performed on a Perkin Elmer Lambda 2 spectrometer (λ: 200–600 nm), by using quartz cells with a 1 cm or 1 mm optical path at 298 K.

#### Synthetic procedures

Hydrophilic thioethers **1**–**7** at all primary carbon atoms of γ-CD were synthesized from octakis(6-deoxy-6-iodo)-γ-CD by nucleophilic displacement reaction with sulfur nucleophiles by using standard procedures described previously [38].

#### Phase-solubility investigations

Solubility measurements of C_60_ in the presence of γ-CD and γ-CD derivatives in water were carried out according to the method proposed by Higuchi and Connors [40]. In glass vials containing excess amounts of C_60_, aqueous solutions of γ-CD or γ-CD derivatives with different concentrations were added. The vials were sealed, protected from light, and magnetically stirred at room temperature for seven days. The solid residues were removed by filtration with syringe filter. According to the Lambert–Beer law, the concentrations of C_60_ in pure water and in CDs solutions were determined from UV–vis extinctions at the absorption maxima (log ε = 4.717, λ_max_ = 335 nm) [18].

#### Procedures for the solubilization of C_60_

Several procedures were employed for the solubilization of C_60_. **Procedure a:** C_60_ stirred in water in the presence of 6 mM γ-CD thioether at rt for 7 d. **Procedure b** was modified from a previously described method [21]: heated under reflux in water/toluene (v/v 1:1) for 3 d (γ-CD thioether concentration 6 mM), and then the resulting mixture dissolved in water after evaporation of the solvents. **Procedure c** was taken from a previous paper [42]: C_60_ stirred in DMF/toluene (v/v 1:1) at rt for 7 d with 6 mM γ-CD thioether, and then the obtained inclusion complex dissolved in water after evaporation of the solvents. **Procedure d:** C_60_ stirred in water/CS_2_ (v/v 1:1) at rt for 7 d with 6 mM γ-CD thioether, and then the obtained inclusion complex dissolved in water after evaporation of the solvents. *n*C_60_ was prepared following a method similar to that reported by Deguchi et al. (**procedure e**) [16]. ***n*C_60_:** A saturated solution of C_60_ in THF was prepared by adding an excess amount of solid C_60_ (>2 mg) into THF (20 mL) and stirring overnight under a nitrogen atmosphere at room temperature. Excess solid was filtered off with a syringe filter. Saturated C_60_/THF solution (500 mL) was placed in a flask and an equal volume of water was added at a rate of ca. 25 mL/min under vigorous stirring. A rotary evaporator was used to remove THF by using a stepwise evaporation approach. The start temperature was set at 30 °C. When the mixture volume had decreased to 500 mL, the temperature was increased at 1 °C/min to 70 °C and maintained at 70 °C until the volume had decreased to 250 mL, at which time an additional 250 mL volume of water was added. The last step was repeated once. The resulting *n*C_60_ solution could be diluted by adding water or concentrated by evaporation as needed.

#### Isothermal kinetic measurement

The procedure for performing isothermal kinetic investigations was modified from a previously described method [37]: A solution of C_60_ in chloroform (ca. 0.5 mg/mL, 0.5 mL) was carefully evaporated in a 1 cm quartz cell under nitrogen flow. After the addition of 3 mL of an aqueous solution containing the necessary amount of γ-CD derivative, the cell was sealed, protected from light, and maintained at 50 °C under gentle shaking. The UV–vis spectra of the resultant solution at appropriate time intervals were measured directly.

#### Dynamic light scattering measurement

Particle size distributions of the aqueous solution or dispersions of C_60_ were determined by dynamic light scattering (DLS) with a ZetaSizer Nano ZS (Malvern Instruments Ltd., Malvern, United Kingdom). From the diffusion coefficient the radius of the particle was determined via the Stokes–Einstein equation. The samples were filtered through a 0.45 μm syringe filter prior to particle-size measurements. Both intensity and volume size distribution curves were calculated from the scattering data by using the software of the instrument.

## Supporting Information

File 1 Detailed dissolution kinetics.
