# Supplementary material for: Molecular solubilization of fullerene C60 in water by γ-cyclodextrin thioethers
Source: Beilstein J Org Chem. 2012 Sep 28;8:1644–51. doi: 10.3762/bjoc.8.188 (PMC3510997; doi:10.3762/bjoc.8.188)
Supplement: File 1 — Detailed dissolution kinetics. [file Beilstein_J_Org_Chem-08-1644-s001.pdf]

Supporting Information

for

Molecular solubilization of fullerene C_{60} in water by γ-cyclodextrin thioethers

Hai Ming Wang and Gerhard Wenz*

Address: Organische Makromolekulare Chemie, Saarland University, Campus Geb. C4.2, D-66123, Saarbrücken, Germany

Email: Prof. Dr. Gerhard Wenz* - g.wenz@mx.uni-saarland.de

* Corresponding author

Detailed dissolution kinetics

2-step model

$$G_{solid} \rightleftarrows G \underset{-CD,\,k_{-1}}{\overset{+CD,\,k_1}{\rightleftarrows}} G\cdot CD \underset{-CD,\,k_{-2}}{\overset{+CD,\,k_2}{\rightleftarrows}} G\cdot CD_2$$

Approximations:

concentrations: [G] = const = [G]$_0$, [CD] = const = [CD]$_0$

stationarity of G·CD: [G·CD$_2$] >>[G·CD]≈const

rate determining steps: $k_1[G]_0[CD]_0 << k_2[G\cdot CD][CD]_0$, $k_{-2}[G\cdot CD_2] << k_{-1}[G\cdot CD]$

rate equation: $$\frac{d[G\cdot CD_2]}{dt} = k_1[CD]_0[G]_0 - k_{-2}[G\cdot CD_2]$$

equilibrium state: $$k_1[CD]_0[G]_0 \equiv k_{-2}[G\cdot CD_2]_{equ}$$

equilibrium constant: $$K' = \frac{k_1}{k_{-2}} = \frac{[G\cdot CD_2]_{equ}}{[G][CD]} \cong \frac{[G\cdot CD_2]_{equ}}{[G]_0[CD]_0}$$

rate equation of complexation:

$$\frac{d[G\cdot CD_2]}{dt} = k_1[G]_0[CD]_0 - k_{-2}[G\cdot CD_2] = k_1[G]_0[CD]_0(1 - c'[G\cdot CD_2])$$

with $c' = \frac{1}{[G]_0[CD]_0 K'} = \frac{1}{[G\cdot CD_2]_{equ}}$

integrated form: $$\frac{\ln(1 - c'[G\cdot CD_2])}{c'} = -k_1[G]_0[CD]_0 t$$

kinetics of complexation: $$[G\cdot CD_2] = (1 - \exp(-k_1 c'[G]_0[CD]_0 t))/c'$$

$$[G\cdot CD_2] = [G\cdot CD_2]_{equ}(1 - \exp(-k_{-2}t))$$

initial slope: $$\left(\frac{d[G\cdot CD_2]}{dt}\right)_{t=0} = [G\cdot CD_2]_{equ}k_{-2} = k_1[G]_0[CD]_0$$

1-step model

$$G_{solid} \underset{\longleftarrow}{\overset{\longrightarrow}{}} G \underset{-2CD,\, k_-}{\overset{+2CD,\, k}{\rightleftarrows}} G \cdot CD_2$$

Approximations:

concentrations: $[G]$ = const = $[G]_0$, $[CD]$ = const = $[CD]_0$

rate equation: $$\frac{d[G\cdot CD_2]}{dt} = k[CD]_0^2[G]_0 - k_-[G\cdot CD_2]$$

equilibrium state: $$k[CD]_0^2[G]_0 \equiv k_-[G\cdot CD_2]_{equ}$$

equilibrium constant: $$K = \frac{k}{k_-} = \frac{[G\cdot CD_2]_{equ}}{[G][CD]^2} \cong \frac{[G\cdot CD_2]_{equ}}{[G]_0[CD]_0^2}$$

Rate equation of complexation:

$$\frac{d[G\cdot CD_2]}{dt} = k[G]_0[CD]_0^2 - k_-[G\cdot CD_2] = k[G]_0[CD]_0^2(1 - c[G\cdot CD_2])$$

with $c = \frac{1}{[G]_0[CD]_0^2 K} = \frac{1}{[G\cdot CD_2]_{equ}}$

integrated form: $$\frac{\ln(1-cx)}{c} = -k[CD]_0^2[G]_0\, t$$

Kinetics of complexation: $$[G\cdot CD_2] = (1 - \exp(-kc[G]_0[CD]_0 t))/c$$

$$[G\cdot CD_2] = [G\cdot CD_2]_{equ}(1 - \exp(-k_- t))$$

Initial slope: $$\left(\frac{d[G\cdot CD_2]}{dt}\right)_{t=0} = [G\cdot CD_2]_{equ}k_- = k[G]_0[CD]_0^2$$
